# Optimising prediction of mortality, stroke, and major bleeding for patients with atrial fibrillation: validation of the GARFIELD-AF tool in UK primary care electronic records

**DOI:** 10.3399/BJGP.2023.0082

**Published:** 2023-10-17

**Authors:** Patricia N Apenteng, David Prieto-Merino, Siew Wan Hee, Trudie CA Lobban, Rishi Caleyachetty, David A Fitzmaurice

**Affiliations:** Institute of Applied Health Research, University of Birmingham, Birmingham, UK; Warwick Medical School, University of Warwick, Coventry, UK.; Faculty of Medicine, University of Alcala, Madrid, Spain.; Warwick Medical School, University of Warwick, Coventry, UK.; AF Association and Arrhythmia Alliance, Winchester, UK.; Warwick Medical School, University of Warwick, Coventry, UK.; Warwick Medical School, University of Warwick, Coventry, UK.

**Keywords:** all-cause mortality, anticoagulation, atrial fibrillation, bleeding, risk stratification, stroke

## Abstract

**Background:**

The GARFIELD-AF tool is a novel risk tool that simultaneously assesses the risk of all-cause mortality, stroke or systemic embolism, and major bleeding in patients with atrial fibrillation (AF).

**Aim:**

To validate the GARFIELD-AF tool using UK primary care electronic records.

**Design and setting:**

A retrospective cohort study using the Clinical Practice Research Datalink (CPRD) linked with Hospital Episode Statistics data and Office for National Statistics mortality data.

**Method:**

Discrimination was evaluated using the area under the curve (AUC) and calibration was evaluated using calibration-in-the-large regression and calibration plots.

**Results:**

A total of 486 818 patients aged ≥18 years with incident diagnosis of non-valvular AF between 2 January 1998 and 31 July 2020 were included; 50.6% (*n* = 246 425/486 818) received anticoagulation at diagnosis The GARFIELD- AF models outperformed the CHA_2_DS_2_VASc and HAS-BLED scores in discrimination ability of death, stroke, and major bleeding at all the time points. The AUC for events at 1 year for the 2017 models were: death 0.747 (95% confidence interval [CI] = 0.744 to 0.751) versus 0.635 (95% CI = 0.631 to 0.639) for CHA_2_DS_2_VASc; stroke 0.666 (95% CI = 0.663 to 0.669) versus 0.625 (95% CI = 0.622 to 0.628) for CHA_2_DS_2_VASc; and major bleeding 0.602 (95% CI = 0.598 to 0.606) versus 0.558 (95% CI = 0.554 to 0.562) for HAS- BLED. Calibration between predicted and Kaplan– Meier observed events was inadequate with the GARFIELD-AF models.

**Conclusion:**

The GARFIELD-AF models were superior to the CHA_2_DS_2_VASc score for discriminating stroke and death and superior to the HAS-BLED score for discriminating major bleeding. The models consistently underpredicted the level of risk, suggesting that a recalibration is needed to optimise its use in the UK population.

## INTRODUCTION

Oral anticoagulation (OAC) substantially reduces the risk of AF-related stroke.^1^ However, OAC increases the risk of bleeding, and AF management guidelines recommend the use of risk stratification tools to guide decisions on anticoagulation.^2^^,^^3^ The European Society of Cardiology and the National Institute for Health and Care Excellence (NICE) AF guidelines recommend the CHA_2_DS_2_VASc score for assessing stroke risk.^3^ Until recently, both guidelines recommended the HAS-BLED score for accessing bleeding risk; however, since 2021 NICE has recommended the ORBIT-AF risk score.^3^

The recommended scores are widely used in clinical practice, nevertheless up to 15% of patients with AF at risk of stroke in England do not receive guideline- recommended therapy.^4^ The GARFIELD-AF risk tool is a novel risk tool that simultaneously assesses a patient’s risk of mortality, stroke or systemic embolism, and risk of major bleeding.^5^^,^^6^ The GARFIELD-AF tool was developed based on 39 898 patients enrolled on the GARFIELD- AF registry in 2017,^5^ and a new version published in 2021 predicted events up to 2 years from diagnosis.^6^ Initial evaluations indicate that both versions are superior to CHA_2_DS_2_VASc in predicting ischaemic stroke/systemic embolism and superior to HAS-BLED in predicting bleeding risk.^5^^,^^6^

GARFIELD-AF is an international prospective observational study of patients aged ≥18 years with newly diagnosed AF and ≥1 investigator-determined risk factor for stroke.^7^^,^^8^ There were a total of 52 080 participants enrolled in 35 countries who were followed for a minimum of 2 years; 3574 of the GARFIELD-AF cohort were recruited in the UK.^9^ The GARFIELD-AF tool can potentially be embedded into primary care electronic systems to aid decision making regarding anticoagulation so that patients who require anticoagulation receive it and those that do not need it do not receive it.

The performance of the prediction model tends to vary across settings and populations, and external validation is required to fully appreciate the generalisability of a prediction model.^10^^,^^11^ The purpose of this study was to validate the GARFIELD-AF tool in patients with AF in an NHS primary care electronic health records database, and compare the performance of the GARFIELD-AF tool with the CHA_2_DS_2_VASc and HAS-BLED scores.

**Table:** How this fits in

Anticoagulation reduces the risk of atrial fibrillation (AF)-related stroke at the cost of an increased risk of bleeding. The CHA_2_DS_2_VASc score is used to assess stroke risk in patients with AF, whereas the HAS- BLED or ORBIT-AF scores are used to assess bleeding risk. A novel tool, GARFIELD-AF simultaneously predicts the risk of stroke death and bleeding in patients with AF; however, its performance has not been tested in the UK population. The GARFIELD-AF models had better discriminatory ability than the CHA_2_DS_2_VASc and HAS-BLED scores in the UK population; however, it underestimated the level of risk.

## METHOD

### Source of data

The primary data source was the Clinical Practice Research Datalink (CPRD), an electronic primary care database comprising anonymised patient medical records from GPs, with coverage of over 19 million patients from 738 practices in the UK.^12^ Data were extracted by CPRD and linked with Hospital Episode Statistics (HES) data, which provides information on all hospital admissions, and mortality data from the Office for National Statistics (ONS).

### Study population

The study population was defined as adults aged ≥18 years with incident diagnosis of non-valvular AF between 2 January 1998 and 31 July 2020, and eligible for linkage with HES and ONS data.

### Follow up

Start of follow up was defined as the recorded date the patient was diagnosed with non-valvular AF. End of follow up was defined as death as recorded by ONS, end of practice registration, or last collection date, whichever occurred first.

### Covariates

The covariates for the GARFIELD-AF models are: age, sex, pulse, systolic blood pressure and diastolic blood pressure, weight, height, ethnicity, current smoking, and paroxysmal AF; history of vascular disease, diabetes, cirrhosis, peripheral vascular disease, stroke, bleeding, heart failure, chronic kidney disease, sleep apnoea, dementia, and/or carotid occlusive disease; and anticoagulant use and antiplatelet use. The covariates and coefficients for the 2017 and 2021 models are detailed in Supplementary Tables S1 and S2.

The main difference between the 2017 and the 2021 models is that the 2021 models have a wider range of variables. For example, the 2017 GARFIELD-AF model for stroke includes the variables age, history of stroke, bleeding, heart failure, chronic kidney disease, region, ethnicity, and anticoagulant use. The 2021 GARFIELD- AF model for stroke has the additional variables female sex, history of carotid occlusive disease, dementia, and smoking. For the GARFIELD-AF 2017 models for death, there exists a full version and a simpler version that comprises a reduced set of variables (age, pulse, systolic blood pressure, history of vascular disease, history of bleeding, heart failure, renal disease, and anticoagulant use), whereas the 2021 death model has just one version.

The covariates for the CHA_2_DS_2_VASc score are: history of congestive heart failure, hypertension, age, diabetes, prior stroke, vascular disease, and sex. The covariates for the HAS-BLED score are: hypertension, abnormal liver or renal function, history of stroke, bleeding history, labile international normalised ratio, age, drug use at time of diagnosis (antiplatelets or non-steroidal anti-inflammatory drugs), or alcohol use.

The baseline variables for the GARFIELD- AF models and CHA_2_DS_2_VASc and HAS-BLED scores were defined from CPRD data using Medical Code IDs. Details are provided in Supplementary Box S1.

### Definition of endpoints

The study endpoints were all-cause mortality; ischaemic stroke/systemic embolism, defined as the combined endpoint of any ischaemic stroke, transient ischaemic attack, or systemic embolism; and major bleeding (including haemorrhagic stroke), defined as bleeding requiring admission to hospital. The first occurrence of an ischaemic stroke/systemic embolism after AF diagnosis was the endpoint for ischaemic stroke/systemic embolism, and the first occurrence of major bleeding after AF diagnosis was the endpoint for major bleeding.

### Outcome variables

Outcome variables were defined from both Medical Code IDs and International Classification of Diseases 10th Revision codes for HES and ONS mortality data, as detailed in Supplementary Box S2.

### Statistical analysis

The GARFIELD-AF models were applied to the CPRD dataset to obtain the predicted risks for each outcome. The performance of the tool was measured in terms of calibration using calibration-in-the-large regression and calibration plots, and in terms of discrimination using the area under the receiver operating characteristic curve (AUC), also referred to as the C-statistic. The performance of the models was compared with the CHA_2_DS_2_VASc and HAS- BLED scores by comparing the AUC of each model. The CHA_2_DS_2_VASc score, in addition to predicting the risk of stroke in patients with AF, has been shown to predict mortality in patients with several diseases, regardless of the presence of AF.^13^ The performance of the CHA_2_DS_2_VASc score for predicting stroke and death was compared with the GARFELD-AF models for stroke and death, and the performance of the HAS-BLED score for predicting bleeding was compared with the GARFIELD-AF bleeding models. The treatment effect was estimated by running separate Cox regression models for each outcome (death, stroke, and bleeding) and adjusting each model for all the variables that contribute to the GARFIELD-AF 2021 score for that outcome.

Each variable was assessed for the degree of missingness. The assessment for discrimination and calibration was performed on the whole dataset and repeated in patients without missing data in any score. Subgroup analysis was conducted according to risk stratification of stroke (high, moderate, and low according to CHA_2_DS_2_VASc) and bleeding (HAS- BLED <2 or >2), and for individuals receiving anticoagulation or no anticoagulation at baseline.

## RESULTS

**Figure 1.**
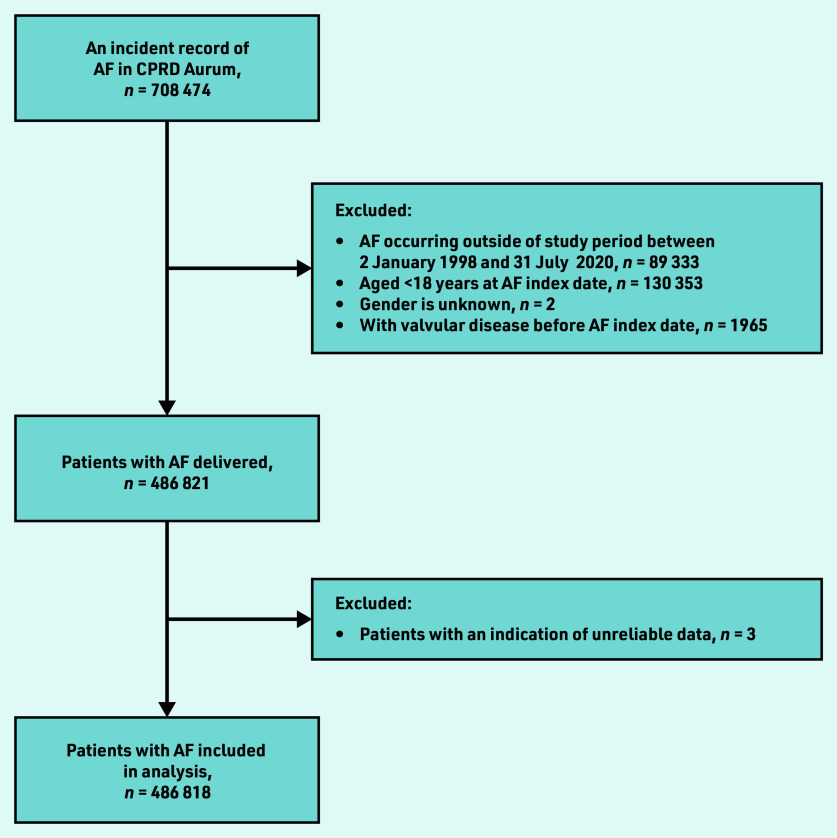
*Flowchart of derivation of CPRD cohort. AF = atrial fibrillation. CPRD = Clinical Practice Research Datalink.*

A total of 708 474 patients had an incident record of AF in CPRD Aurum. Of these, 486 818 met the inclusion criteria for the study (Figure 1). The median follow up was 3.975 years (interquartile range 1.6– 7.7; minimum 0 to maximum 22.6).

### Baseline characteristic of participants

The baseline characteristics for the CPRD validation cohort, the UK GARFIELD-AF subcohort, and the global GARFIELD- AF cohort are presented in Table 1. The participants in the UK cohorts were older compared with the global GARFIELD- AF cohort (mean age 75 years versus 70 years). The UK cohorts were predominantly of White ethnicity (95.2% [*n* = 447 972/470 743] CPRD and 98.8% [*n* = 3441/3483] GARFIELD-AF UK versus 63.1% [*n* = 32 028/50 976] global cohort) and had a higher prevalence of history of bleeding (6.8% [*n* = 33 205/486 818] CPRD versus 2.5% [*n* = 1318/52 080] global cohort).

**Table 1.** Baseline characteristics of the CPRD validation cohort, the UK GARFIELD-AF cohort, and the global GARFIELD-AF cohort

**Variable**	**Validation cohort, *n* = 486 818**	**GARFIELD-AF UK, *n* = 3574**	**Global GARFIELD-AF, *n* = 52 080**
**Age, years, mean (SD)**	74.6 (12.2)	74.5 (9.5)	69.7 (11.5)

**Age group, years, *n* (%)**			
<65	89 281 (18.3)	471 (13.2)	15 708 (30.2)
65–74	123 661 (25.4)	1178 (33.0)	16 960 (32.6)
≥75	273 876 (56.3)	1925 (53.9)	19 412 (37.3)

**Female, *n* (%)**	227 370 (46.7)	1522 (42.6)	23 011 (44.2)

**Ethnicity, *n* (%)**	470 743	3483	50 796
White	447 972 (95.2)	3441 (98.8)	32 028 (63.1)
Asian	7373 (1.6)	13 (0.4)	14 302 (28.2)
Black/mixed/other	15 398 (3.3)	29 (0.8)	4466 (8.8)

**Clinical observations at diagnosis, mean (SD)**			
Pulse	79.2 (18.9)	87.5 (22.7)	90.4 (26.7)
Systolic blood pressure	134.8 (19.6)	133.0 (17.7)	133.5 (19.8)
Diastolic blood pressure	77.4 (11.7)	77.0 (11.3)	79.7 (12.9)
Weight, kg	81.3 (21.0)	83.6 (19.7)	77.6 (19.0)
Height, m	1.68 (0.1)	1.69 (0.10)	1.67 (0.10)
BMI	28.8 (6.3)	29.2 (6.2)	27.8 (5.7)

**Medical history, *n* (%)**			
Congestive heart failure	33 817 (6.9)	274 (7.7)	11 758 (22.6)
History of hypertension	344 590 (70.8)	2483 (69.5)	39 643 (76.1)
Diabetes mellitus	66 876 (13.7)	629 (17.6)	11 555 (22.2)
Prior stroke/TIA	52 103 (10.7)	450 (12.6)	5961 (11.4)
Vascular disease	41 523 (8.5)	760 (21.3)	7682 (14.8)
Peripheral vascular disease	10 318 (2.1)	—	—
Carotid occlusive disease	2107 (0.4)	52 (1.5)	1545 (3.0)
History of bleeding	33 205 (6.8)	109 (3.0)	1318 (2.5)
Chronic kidney disease (grade ≥3)	69 676 (14.3)	896 (25.1)	5360 (10.3)
Chronic renal failure	7831 (1.6)	—	—
Cirrhosis	1484 (0.3)	11 (0.3)	295 (0.6)
Current smoker	30 168 (6.2)	245 (6.9)	5204 (10.0)
Sleep apnoea	5048 (1.0)	—	—
Dementia	10 830 (2.2)	28 (0.8)	764 (1.5)
Type of AF diagnosed is paroxysmal	65 474 (13.4)	651 (18.2)	14 315 (27.5)
Antiplatelets or NSAIDs use	192 271 (39.5)	—	—
≤8 units alcohol/week	362 641 (74.5)	—	—
>8 units alcohol/week	34 513 (7.1)	—	—

**Risk scores**			
CHA_2_DS_2_VASc score, mean (SD)	2.96 (1.5)	3.3 (1.5)	3.2 (1.6)
CHA_2_DS_2_VASc score categories, *n* (%)	486 818	3528	51 408
0	40 052 (8.2)	62 (1.8)	1516 (2.9)
1	41 135 (8.4)	316 (9.0)	6369 (12.4)
2	88 455 (18.2)	659 (18.7)	10 230 (19.9)
3	126 160 (25.9)	972 (27.6)	12 138 (23.6)
4	124 665 (25.6)	848 (24.0)	11 022 (21.4)
5	51 858 (10.7)	398 (11.3)	5895 (11.5)
≥6	14 723 (3.0)	273 (7.7)	4238 (8.2)
HAS-BLED score,^a^,^b^ mean (SD)	1.62 (0.9)	1.7 (0.9)	1.4 (0.9)
HAS-BLED score categories,^a^ *n* (%)	324 520	2530	37 549
0	30 770 (9.5)	160 (6.3)	5471 (14.6)
1	125 541 (38.7)	941 (37.2)	16 169 (43.1)
2	113 120 (34.9)	950 (37.5)	11 692 (31.1)
3	45 954 (14.2)	391 (15.5)	3570 (9.5)
≥4	9135 (2.8)	88 (3.5)	647 (1.7)

**Treatment at diagnosis, *n* (%)**	486 818	3564	51 354
NOAC	106 994 (22.0)	688 (19.3)	14 129 (27.5)
VKA	141 200 (29.0)	1656 (46.5)	20 206 (39.3)
OAC	246 425 (50.6)	2344 (65.8)	34 335 (66.9)
AP	187 962 (38.6)	1189 (33.4)	18 121 (35.3)

a *The risk factor labile international normalised ratio is not included in the HAS-BLED score. As a result, the maximum HAS-BLED score at baseline is 8 points (not 9).*

b *Denominators of the medical history risk factors vary depending on how many individuals had the information available. The percentages are calculated based on the number of people with information in each risk factor (not shown). AF = atrial fibrillation. AP = antiplatelet. BMI = body mass index. CPRD = Clinical Practice Research Datalink. NOAC = non- vitamin K antagonist oral anticoagulant. NSAID = non- steroidal anti-inflammatory drug. OAC = oral anticoagulation. SD = standard deviation. TIA = transient ischaemic attack. VKA = vitamin K antagonist.*

Four-fifths of the CPRD cohort had CHA_2_DS_2_VASc ≥2, 8.4% had CHA_2_DS_2_VASc = 1, and 8.2% had CHA_2_DS_2_VASc = 0. A total of 50.6% of the CPRD cohort received anticoagulation at diagnosis compared with 65.8% in the UK GARFIELD-AF cohort and 66.9% in the global GARFIELD-AF cohort. Overall, the CPRD cohort had a lower mean CHA_2_DS_2_VASc score compared with the GARFIELD-AF UK and global cohorts: 2.96 (standard deviation [SD] 1.5) versus 3.3 (SD 1.5) and 3.2 (SD 1.6), respectively. In the CPRD cohort 83.0% of the patients with a HAS-BLED score <3 compared with 81.1% in GARFIELD-AF UK and 88.8% in the global GARFIELD-AF cohort (Table 1).

### Missing data in CPRD

There were no missing data in the covariates needed to calculate CHA_2_DS_2_VASc, but 33.3% (*n* = 162 298/486 818) of patients had missing data for calculating HAS-BLED. For the 2017 GARFIELD-AF models there were no missing data for the predictors for the bleeding model, but 16 075 patients (3.3%) had missing data for the stroke model and 65.7% (*n* = 319 621/486 818) had missing data for the mortality model. Therefore, it was only possible to calculate all three models (bleeding, stroke, and mortality) in 164 427 patients (33.7%). For the 2021 GARFIELD- AF models, 65.1% (*n* = 316 695/486 818) of patients had missing data for the bleeding model, 69.4% (*n* = 337 713/486 818) had missing data for the stroke model, and 89.1% (*n* = 433 590/486 818) had missing data for the mortality model. There were 53 228 from the 486 818 patients (10.9%) who had complete data for all three models (bleeding, stroke, and mortality).

### External validation for the GARFIELD-AF models

Table 2 shows the full data for the 2017 1-year mortality, stroke, and bleeding models and the 2021 models (each model with 1-month, 1-year, and 2-year follow up).

**Table 2.** Predicted and Kaplan–Meier estimated risks for the GARFIELD-AF models

**Year of GARFIELD-AF model**	**Outcome**	**Months of follow-up, *n***	**Patients with predicted risk, *n***	**Average predicted risk, %**	**KM, %**	**N0, *n***	**P0, %**	**N1, *n***	**P1, %**	**AUC (95% CI)**
2017	Death	12	167 197	4.51	11.89	130 871	4.03	19 110	8.16	0.748 (0.744 to 0.751)
2017 (full)	Death	12	107 404	6.81	10.81	84 574	6.09	11 132	12.76	0.748 (0.743 to 0.752)
2017	Stroke	12	470 743	1.65	8.29	357 601	1.48	37 466	2.32	0.666 (0.663 to 0.669)
2017	Bleed	12	486 818	1.40	6.32	377 482	1.31	28 521	1.61	0.603 (0.599 to 0.606)
2021	Death	1	53 228	0.81	1.55	52 218	0.79	823	1.71	0.753 (0.737 to 0.769)
2021	Stroke	1	149 105	0.22	4.27	140 940	0.22	6358	0.29	0.622 (0.615 to 0.629)
2021	Bleed	1	170 123	0.22	1.60	164 732	0.21	2706	0.25	0.576 (0.565 to 0.586)
2021	Death	12	53 228	5.98	11.82	41 652	5.41	6046	10.64	0.728 (0.722 to 0.735)
2021	Stroke	12	149 105	1.58	8.28	111 751	1.43	11 795	2.17	0.670 (0.665 to 0.676)
2021	Bleed	12	170 123	1.55	7.25	123 287	1.48	11 250	1.85	0.598 (0.593 to 0.604)
2021	Death	24	53 228	10.27	19.64	32 016	8.89	9445	17.02	0.731 (0.726 to 0.737)
2021	Stroke	24	149 105	2.62	11.10	89 415	2.28	14 925	3.56	0.683 (0.678 to 0.687)
2021	Bleed	24	170 123	2.41	11.66	91 111	2.23	16 425	2.81	0.602 (0.598 to 0.607)

*AUC = area under the curve (C-statistic). Full = full version of the 2017 death model. KM = estimated risk using Kaplan–Meier method. N0 = number of patients without the outcome at end of follow up. P0 = average predicted risk in patients in N0. N1 = number of patients with positive outcome at end of follow up. P1 = average predicted risk in patients in N1.*

### Discrimination

The AUCs in Table 2 range from 0.576 (95% CI = 0.565 to 0.586) for the 2021 model for bleeding in 1 month to 0.753 (95% CI = 0.737 to 0.769) for the 2021 model for death in 1 month. At 1-year follow up the 2017 and 2021 models performed very similarly for the outcomes of stroke (AUCs 0.666 versus 0.670, respectively) and bleeding (AUCs 0.603 versus 0.598, respectively), with overlapping CIs in both outcomes. The 1-year 2017 model slightly, but significantly, outperformed the 1-year 2021 model for predicting death with AUCs 0.748 (95% CI = 0.743 to 0.752) and 0.728 (95% CI = 0.722 to 0.735), respectively, with non-overlapping CIs.

### Calibration

**Figure 2.**
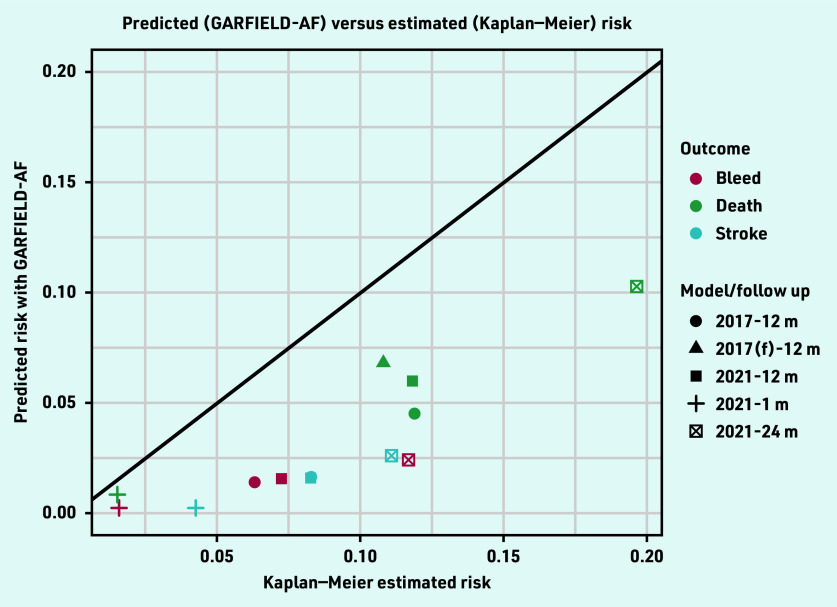
*Predicted versus Kaplan–Meier risk of the GARFIELD-AF models. f = full. m = month.*

**Figure 3.**
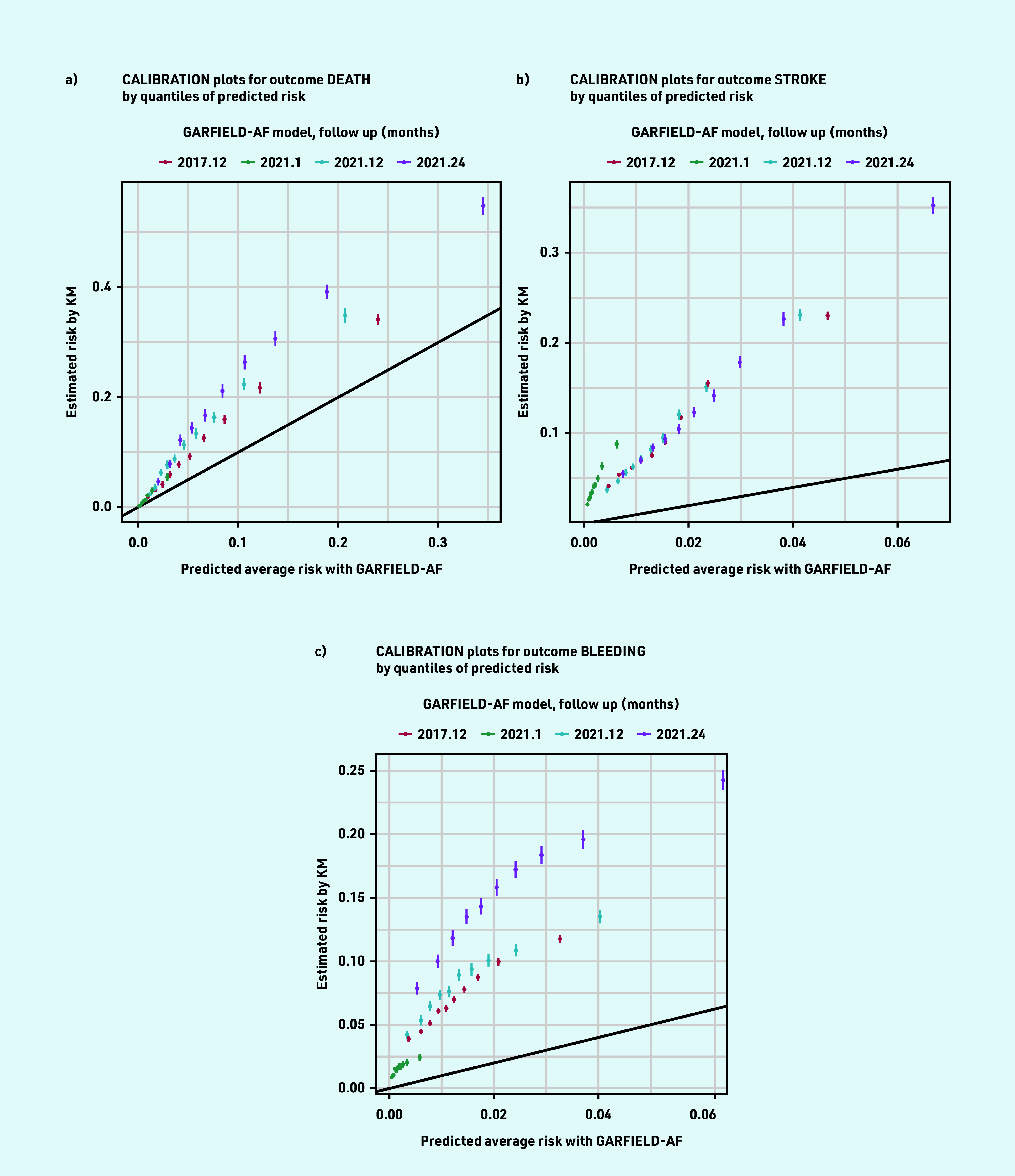
*Calibration plots for death, stroke, and bleeding outcomes by quantiles of predicted risk. KM = Kaplan—Meier.*

For the three outcomes, both the 2017 and 2021 GARFIELD-AF models consistently predicted less average risk than the observed risks in the population estimated using the Kaplan–Meier method (Table 2 and Figure 2). The 2017 tool performed slightly better than the 2021 tool at 1-year follow up for the three outcomes. The calibration plots show that the differences between the GARFIELD-AF’s predicted risks and the Kaplan–Meier estimated risks grow in the larger quintiles (Figure 3).

### Comparison with GARFIELD-AF models and CHA_2_DS_2_VASc and HAS-BLED scores

The GARFIELD-AF models consistently outperformed the CHA_2_DS_2_VASc and HAS- BLED scores (Table 3). The AUC for the 2017 models at 1-year follow up were: death 0.748 versus 0.635 for CHA_2_DS_2_VASc, stroke 0.666 versus 0.625 for CHA_2_DS_2_VASc, and bleeding 0.602 versus 0.558 for HAS- BLED. The AUC for the 2021 models at 1 year were death: 0.728 versus 0.616 for CHA_2_DS_2_VASc, stroke 0.670 versus 0.620 for CHA_2_DS_2_VASc, and bleeding 0.604 versus 0.560 for HAS-BLED. *P*-values were <0.0001 with non-overlapping CIs in all comparisons.

**Table 3.** Comparison of GARFIELD-AF models with CHAD and HASB

**Year of GARFIELD-AF model**	**Other**	**Outcome**	**Follow up, months**	**AUC of the GARFIELD-AF model in the row (95% CI)**	**AUC of the other model in the row (95% CI)**	***P*-value comparing AUCs**
2017	CHAD	Death	12	0.748 (0.744 to 0.751)	0.635 (0.631 to 0.639)	<0.0001
2017 (full)	CHAD	Death	12	0.748 (0.743 to 0.752)	0.627 (0.622 to 0.632)	<0.0001
2017	CHAD	Stroke	12	0.666 (0.663 to 0.669)	0.625 (0.622 to 0.628)	<0.0001
2017	HASB	Bleed	12	0.602 (0.598 to 0.606)	0.558 (0.554 to 0.562)	<0.0001
2021	CHAD	Death	1	0.753 (0.737 to 0.769)	0.609 (0.591 to 0.627)	<0.0001
2021	CHAD	Stroke	1	0.622 (0.615 to 0.629)	0.588 (0.581 to 0.595)	<0.0001
2021	HASB	Bleed	1	0.584 (0.571 to 0.596)	0.549 (0.538 to 0.561)	<0.0001
2021	CHAD	Death	12	0.728 (0.722 to 0.735)	0.616 (0.609 to 0.623)	<0.0001
2021	CHAD	Stroke	12	0.670 (0.665 to 0.676)	0.620 (0.615 to 0.625)	<0.0001
2021	HASB	Bleed	12	0.604 (0.598 to 0.611)	0.560 (0.554 to 0.566)	<0.0001
2021	CHAD	Death	24	0.731 (0.726 to 0.737)	0.625 (0.619 to 0.631)	<0.0001
2021	CHAD	Stroke	24	0.683 (0.678 to 0.687)	0.634 (0.630 to 0.639)	<0.0001
2021	HASB	Bleed	24	0.607 (0.602 to 0.613)	0.559 (0.554 to 0.565)	<0.0001

*AUC = area under the curve (C-statistic). CHAD = CHA_2_DS_2_VASc. HASB = HAS-BLED.*

### Subgroup analyses

Patients not taking OAC showed a higher average risk of events in almost every version of the model than those patients taking OAC. The AUC was always larger in patients not taking OAC. This is compatible with OAC lowering the risks of patients and making it more difficult to tell who is going to have an event (lower AUC) (see Supplementary Table S3).

After adjusting for the GARFIELD-AF 2021 risk factors in Cox regression models, anticoagulation had a protective effect from death with an adjusted hazard ratio (aHR) 0.58 (95% CI = 0.50 to 0.68), a protective effect for stroke with an aHR 0.71 (95% CI = 0.63 to 0.81), and a non-significant protective effect on bleeding with an aHR 0.90 (95% CI = 0.76 to 1.05) (data not shown).

When stratified according to risk levels, the GARFIELD-AF models performed better in patients at high risk compared with moderate risk for stroke according to CHA_2_DS_2_VASc (see Supplementary Table S3). The AUCs for 2017 1-year risk for the stroke model were: high risk 0.652 (95% CI = 0.649 to 0.656), moderate risk 0.559 (95% CI = 0.545 to 0.572), and low risk 0.526 (95% CI = 0.508 to 0.543).

### Complete-case analysis

The data for the complete-case analysis are shown in Supplementary Table S4. When analysis was restricted to the 30 666 patients with data to calculate all scores the AUCs for the 2017 model were: death 0.719 (95% CI = 0.710 to 0.728), stroke 0.677 (95% CI = 0.665 to 0.689), and bleeding 0.589 (95% CI = 0.573 to 0.598), indicating a similar performance to the whole group analysis for stroke and bleeding, and a slight difference for death. Like in the main analyses, the models were miscalibrated when restricted to cases with patients with a full dataset, showing important differences between the GARFIELD-AF- predicted and Kaplan– Meier estimated risks (see Supplementary Figure S1).

## DISCUSSION

### Summary

In this study population of 486 818 patients with incident AF, the GARFIELD-AF models have good discrimination for predicting death and moderate discrimination for predicting stroke and bleeding, but consistently below the discriminations reported in the original GARFIELD- AF publications. The current findings show that the models are superior to the CHA_2_DS_2_VASc score for predicting stroke and the HAS-BLED score for predicting bleeding. However, all versions of the models consistently underpredicted the level of risk. There were no significant differences in the performance of the 2017 and 2021 models at 1 year for bleeding and stroke but the 2017 tool showed a slightly better performance for death.

### Strengths and limitations

The study has several strengths, the data source CPRD is a primary care database representing approximately 10% of the UK primary care population and provided a large real-world sample of patients with incident AF with good statistical power. Linkage with HES and ONS data improved the robustness of the data, reducing the chance of missing out any of the outcomes of interest.

A rigorous process of codelist development was used in the study, with a primary care clinician overseeing and reviewing all the codelists. There was a significant amount of missing data for the 2021 models and the 2017 full-death model. The volume of missing data was too large for multiple imputation; comparison of the whole dataset and complete cases showed little difference.

### Comparison with existing literature

Within the global GARFIELD-AF study population the 2017 tool had a modest predictive ability for stroke (0.69, 95% CI = 0.67 to 0.71) and major bleeding (0.66, 95% CI = 0.62 to 0.69), and a good performance for death (0.77, 95% CI = 0.76 to 0.78).^5^ These values were slighter better when the 2021 models were evaluated in the GARFIELD-AF study population, the AUC at 1 year were: stroke 0.70 (95% CI = 0.68 to 0.72), major bleeding 0.69 (95% CI = 0.67 to 0.71), and death 0.76 (95% CI = 0.75 to 0.77).^6^ In both the 2017 and 2021 internal validation, the models performed better than within the UK CPRD cohort; however, this is what one would expect of an internal validation.

The GARFIELD-AF models performed better in the ORBIT-AF population than in the UK: AUC death 0.75 (95% CI = 0.74 to 0.76), stroke 0.68 (95% CI = 0.64 to 0.71), and major bleeding 0.64 (95% CI = 0.62 to 0.66) for the 2021 model,^6^ and similar outcomes for the 2017 models.^5^

The current study is the first, to the authors’ knowledge, to independently validate the GARFIELD-AF 2021 tool externally; however, the 2017 tool has been previously evaluated. An independent evaluation of the GARFIELD-AF 2017 for stroke and bleeding in the Danish population reported higher discriminatory ability than was found in the current CPRD cohort: stroke (AUC 0.71, 95% CI = 0.70 to 0.72); HAS-BLED AUC 0.64 (95% CI = 0.63 to 0.66) in patients using OAC therapy.^14^ The same study found that the GARFIELD- AF tool was superior to CHA_2_DS_2_VASc but comparable with HAS-BLED, whereas in the current study the population discrimination ability of GARFIELD-AF was superior to both CHA_2_DS_2_VASc and HAS- BLED.^14^ In addition, both the stroke and bleeding models were well calibrated in the Danish cohort.^14^ Another study comparing the 2017 GARFIELD-AF bleeding model to HAS- BLED also found the bleeding model to have modest predictive value, although the C-statistic for GARFIELD-AF was lower than in the current study (0.56, 95% CI = 0.54 to 0.57).^15^

The GARFIELD-AF models consistently underpredicted the level of risk in the CPRD cohort. There may be a number of reasons for this — it may be the impact of geographical variation; there were significant variations in outcomes across countries within the GARFIELD-AF registry even after adjustment for baseline characteristics and antithrombotic treatment.^16^ There were differences in the baseline characteristics in the UK population, notably the UK population were older. Also, the UK GARFIELD-AF population had a higher incidence of stroke, bleeding, and mortality compared with the global population.^9^

### Implications for research and practice

The novelty of the GARFIELD-AF tool is simultaneous prediction of stroke, bleeding, and death. Death has been shown to be an important outcome in AF, prompting recommendations for a more integrated management of patients with AF;^2^ however, there is currently no designated tool for assessing mortality in patients with AF. The death models had the best predictive ability, and the 2017 abridged death model offers a good alternative with a reduced set of predictors that are available in UK primary care records.

The 2017 tool would be better suited to clinical use in the UK because of the better availability of the predictors in primary care records. Recalibration will optimise the use of the GARFIELD-AF tool in the UK population without losing the information captured from the original tool. Incorporating a recalibrated tool into UK primary care electronic systems would help clinicians evaluate the risk–benefit ratio of anticoagulation, and potentially improve risk stratification and decision making regarding anticoagulation for patients with AF.

## Data

Access to CPRD data is subject to protocol approval by an Independent Scientific Advisory Committee. A non-statutory expert advisory body oversees access to linked CPRD data for research purposes.
